# Biomimetic Methacrylated Gelatin Hydrogel Loaded With Bone Marrow Mesenchymal Stem Cells for Bone Tissue Regeneration

**DOI:** 10.3389/fbioe.2021.770049

**Published:** 2021-12-02

**Authors:** Jun Li, Wenzhao Wang, Mingxin Li, Ping Song, Haoyuan Lei, Xingyu Gui, Changchun Zhou, Lei Liu

**Affiliations:** ^1^ Department of Orthopedics, Orthopedic Research Institute, National Clinical Research Center for Geriatrics, West China Hospital, Sichuan University, Chengdu, China; ^2^ National Engineering Research Center for Biomaterials, Sichuan University, Chengdu, China; ^3^ College of Biomedical Engineering, Sichuan University, Chengdu, China

**Keywords:** large segment bone defect, GelMA, hydrogel, BMSCs, ECM

## Abstract

Large-segment bone defect caused by trauma or tumor is one of the most challenging problems in orthopedic clinics. Biomimetic materials for bone tissue engineering have developed dramatically in the past few decades. The organic combination of biomimetic materials and stem cells offers new strategies for tissue repair, and the fate of stem cells is closely related to their extracellular matrix (ECM) properties. In this study, a photocrosslinked biomimetic methacrylated gelatin (Bio-GelMA) hydrogel scaffold was prepared to simulate the physical structure and chemical composition of the natural bone extracellular matrix, providing a three-dimensional (3D) template and extracellular matrix microenvironment. Bone marrow mesenchymal stem cells (BMSCS) were encapsulated in Bio-GelMA scaffolds to examine the therapeutic effects of ECM-loaded cells in a 3D environment simulated for segmental bone defects. *In vitro* results showed that Bio-GelMA had good biocompatibility and sufficient mechanical properties (14.22kPa). A rat segmental bone defect model was constructed *in vivo*. The GelMA-BMSC suspension was added into the PDMS mold with the size of the bone defect and photocured as a scaffold. BMSC-loaded Bio-GelMA resulted in maximum and robust new bone formation compared with hydrogels alone and stem cell group. In conclusion, the bio-GelMA scaffold can be used as a cell carrier of BMSC to promote the repair of segmental bone defects and has great potential in future clinical applications.

## Introduction

Bone defects are serious health problems that cause hundreds of millions of surgical procedures worldwide each year (Fang et al., 2016). Large bone defects are often sequelae of trauma, tumor (osteosarcoma), or congenital disease (Agarwal and García, 2015). Deficiency of blood supply, infection of the bone or surrounding tissue, systemic diseases, and so on can adversely affect bone healing, leading to delayed union or nonunion of the bone (Giannoudis et al., 2016; Zakhary and Thakker, 2017; Biggemann et al., 2018). Autogenous bone transplantation is the gold standard in the treatment of bone defect (Stanovici et al., 2016). However, the lack of bone donors and the high time and cost of surgery have seriously hindered the clinical application. In addition, allografts from genetically different species face immune rejection and high reabsorption rates, leading to associated complications (Larsen et al., 2011; Bez et al., 2017). Various artificial materials, such as polymers, inorganic nonmetallic materials, metal materials, and composites, have been used for bone repair or replacement (Cao et al., 2020). However, none of these approaches can help treat patients economically and effectively (Bose et al., 2012; Garot et al., 2020). Repairing large bone defects remains a huge challenge. Bone tissue engineering has become an interdisciplinary field with great potential for development (Kupikowska-Stobba and Kasprzak, 2021). Bone tissue engineering is using new knowledge-based and cell-friendly materials capable of simulating the structural, mechanical, and biological properties of natural bone (Haleem et al., 2020; Gonçalves et al., 2021). Scaffolds and cells are essential components of bone tissue engineering, and the right combination is expected to provide improved clinical treatment (Moreno Madrid et al., 2019). Scaffolds that mimic the structure and composition of bone tissue, also known as bionic scaffolds, have been extensively studied (Cox et al., 2015; Wade et al., 2015).

Bone tissue consists of osteocytes and extracellular matrix (ECM) (Benmassaoud et al., 2020). The ECM is a reservoir of proteins and proteoglycan, and growth factors (Ravindran and George, 2014; Murshed, 2018). ECM provides a cellular microenvironment that is the basis for mineral phase deposition, bone conduction, and bone induction (Alcorta-Sevillano et al., 2020). Some studies have used ECM as a bone conduction matrix for bone regeneration (Chen et al., 2019). Traditional synthetic, biodegradable polymers have been used to improve the performance of biomaterials (Zhang et al., 2019a; Cui et al., 2020). However, most of these polymers are hydrophobic, limiting their ability to encapsulate cells. Hydrogel is a hydrophilic polymer with inherent three-dimensional structure (Zhao et al., 2021). Gelatin is a protein substance obtained from the hydrolysis of collagen, which has good biocompatibility and biodegradation. Gelatin has fewer aromatic groups, so its immunogenicity is obviously low (Xiao et al., 2019). Gelatins contain arginine–glycine–aspartic acid peptide sequences that promote cell adhesion, proliferation, and differentiation, and are therefore suitable for ECM simulation (Sun et al., 2018). In addition, the matrix metalloproteinase of gelatin can promote cell remodeling and further enhance its biological activity (Xiao et al., 2019). The addition of methacrylic anhydride makes the advantages of gelatin easier to be exploited (Pepelanova et al., 2018). Gelatin methylacrylyl (GelMA), made of gelatin and methacrylic anhydride, is a thermally stable cross-linked hydrogel formed by photoinitiator or ultraviolet irradiation (Xiao et al., 2019). Some studies have shown that GelMA can be used to repair bone defects, deposit extracellular matrix and rich type II collagen, and has a good performance in promoting angiogenesis (Xiang and Cui, 1186). In addition, GelMA can be injected into irregularly shaped bone defects and solidified (Gu et al., 2019). However, GelMA lacks the osteogenic induction capacity required for bone mineralization (Qiao et al., 2020). In most studies, GelMA has been used to repair skull defects, but there has been a lack of research on segmental bone defects, which is one of the most clinically difficult. Mesenchymal stem cell (MSC) is a kind of widely distributed, self-renewing, and differentiated multi-lineage cells (Fu et al., 2019). Bone marrow mesenchymal stem cells (BMSCs) are the most commonly used stem cells in cell therapy and tissue engineering, which can mobilize and migrate from bone marrow to damaged tissue to repair bone and cartilage defects (Zhang et al., 2019b).

In this study, a “soft” and injectable GelMA hydrogel matrix was designed to mimic bone ECM. By rationally controlling the degree of cross-linking density and aperture size, the elastic mechanical properties of GelMA and the mechanical microenvironment of ECM were obtained. Subsequently, BMSC cells were incubated in the hydrogel to test the biocompatibility of the hydrogel *in vitro*. Finally, the *in vivo* repairability of hydrogels was demonstrated in rat models of segmental bone defects. GelMA hydrogel matrix encapsulated with BMSC was an ideal synthetic substitute with excellent osteogenic and angiogenic capabilities.

## Materials and methods

### Animals

In this study, a total of 120 healthy and clean adult female SD rats, aged 4 weeks and weighing 200–250 g, were used and provided by the Experimental Animal Center of West China Clinical Medical College of Sichuan University. Ninety-six of them were only used to construct bone defect models, while the others were used for BMSC isolation and culture. All animal experiments conducted in this study were approved by the animal management and use committee of the West China Clinical Medical College of Sichuan University (approval number: SCXK20150012). The rats were put into a cage 1 week before the experiment to adapt to the environment. Three rats/cage were served with sufficient conventional animal feed, maintaining the room temperature at 21°C, 60% air humidity, and 12-h circadian rhythm.

### Preparation of methacrylated gelatin

The GelMA was synthesized following the procedure described. Briefly, 10 g of gelatin derived from porcine skin was dissolved in 100 ml of PBS in a cleaned Erlenmeyer flask with magnet fish. Then 5 ml of methacrylic anhydride was added very slowly and dropwisely with a syringe pump, and the emulsion was rotated (240 rpm) at 50°C for 2 h and covered with an aluminum foil. Dialysis membrane (Pectro/Por molecular porous membrane tubing, Fisher Scientific, USA) was prepared by cutting them in proper sizes and immersed them into distilled water to soften them. One side was closed by twisting the membrane end and making a knot. The GelMA was transferred with a funnel into the membranes. The second end of the membrane was closed the same way as the first. Membranes were placed into distilled water in a 5-L plastic beaker, and the dialysis was ran at 40°C for 7 days with a magnetic stirrer and covered. The GelMA solution was then quickly and successively filtered with a coffee filter and sterile vacuum Express Plus (0.22 µm) Milipore filtration cup. The sterilized polymer was transferred into 50-ml Falcons and horizontally stored at −80°C for 2 days. The frozen GelMA was lyophilized for 3 days and stored in the dark until use.

### Characterization of methacrylated gelatin hydrogel

The GelMA was dissolved in D_2_O for analysis using 400-Hz nuclear magnetic resonance (Bruker AVANCE AV Ⅱ-400 MHz). The degree of methacrylate substitution was determined by the formula: 1 − (lysine integration signal of GelMA/lysine integration signal of unsubstituted Gelatin) × 100% (Brinkman et al., 2003; Nichol et al., 2010; Loessner et al., 2016). The morphology of the GelMA hydrogel was observed by scanning electron microscope (SEM). Dynamic Mechanical Analyzer (TA Instruments, Q-800, USA) was used to test the storage modulus and loss modulus of the GelMA hydrogel. The rheological properties of the GelMA hydrogel were analyzed by rheometer (MCR302, Anton Paar) (Zhou, 2021).

### Preparation of bone marrow mesenchymal stem cells–methacrylated gelatin hydrogel scaffold

The GelMA solution with a concentration of 5% was prepared by using deionized water, and a photocrosslinking agent (Irgacure 500, BASF Corporation, Germany) with a dosage of 0.25% of GelMA solution (w/v) was added. After mixing the GelMA solution with the photocrosslinking agent evenly, a mixed solution was obtained. The mixed solution was filtered through a 0.22-µm filter membrane and mixed with BMSCs to make the cell suspension. The cell density in the suspension was 2 × 10/ml (Stanovici et al., 2016). The bone defect model was constructed according to the needs, and the PDMS mold of the corresponding size (4 × 4 × 5 cm) was prepared according to the bone defect model. The suspension of the GelMA–BMSCs was added into the PDMS mold, and UV irradiation (*λ* = 365 nm, 40 s) was given. After crosslinking, the GelMA hydrogel bone repair scaffold containing BMSCs was obtained.

### Biocompatibility assessment

The previously prepared BMSC–GelMA hydrogel scaffolds were cultured in normal medium. LIVE/DEAD assay was applied to evaluate the cell viability at 1, 3, 7, and 14 days after culture. The hydrogel scaffolds were washed with PBS and stained with Calcein AM (0.5 μl ml^−1^) and ethidium homodimer-1 (EthD-1, 2 μl/ml) for 2 h at 37°C. The samples were observed under an inverted fluorescence microscope (Nikon, Japan). The number of living and dead cells in the scaffold was counted, and the percentage of living cells in the total number of cells was calculated. Cell Counting Kit-8 (CCK-8, Dojindo, Japan) was used to detect the influence of scaffolds on cell activity on days 1, 4, 7, and 14. A 10-µl CCK8 solution was add to each well of the 96-well plate, incubated at 37°C for 2 h, and the absorbance at 450 nm was measured with a microplate analyzer (Thermo Scientific, Shanghai, China). Each experiment was repeated at least three times.

### Bone defect model construction and stent implantation

SD rats were randomly divided into four groups (16 rats in each group): group A was the model control group, group B was the GelMA hydrogel scaffold group (control group), BMSCs were in group C (control group), and group D was the GelMA hydrogel scaffold containing BMSCs (experimental group). The rats were anesthetized by intraperitoneal injection of pentobarbital, the hair of the left hind limb was cleaned, sterilized with alcohol, and was covered with a sterile dressing. A longitudinal incision was taken from the posterior middle posterior tibia, and the subcutaneous and muscular layers were incised. A 5-mm-long segmental bone defect was created with a bone saw. In group A, the intramedullary nail was used for retrograde fixation. In group B, the GelMA hydrogel scaffold was implanted and fixed with intramedullary nails. BMSC suspension was injected into the bone defect of group C. In group D, BMSC-loaded GelMA hydrogel scaffolds were implanted and fixed with intramedullary nails. The muscle, subcutaneous tissue, and skin were sewn up step by step. At weeks 4 and 8 after surgery, the bone tissue in the bone defect area was taken for histomorphological test, biomechanical property test, and micro-CT test.

### Histomorphological test

Bone tissue was taken from the bone defect area at weeks 4 and 8 after surgery, and hematoxylin–eosin (HE) staining was performed. The isolated specimens were decalcified through ethylene diamine tetraacetic acid (EDTA, Sigma, USA), dehydrated by 80, 90, and 100% ethanol, and embedded in paraffin. The specimens were then cut into 5-μm sections and stained with hematoxylin–eosin (HE), and observed under a BX53 microscope (Olympus, Japan). The new bone and new blood vessels were quantitatively analyzed. Experimental data were expressed as mean ± SD.

### Biomechanical performance test

On the fourth and eighth weeks after surgery, the tibia of the rats was taken for the biomechanical test. The residual soft tissue was removed, and the tibial tip was trimmed to an appropriate length so that the bone defect was located in the middle of the sample. The three-point bending test (Ruige Technology, China) was performed on the biomechanical tester to measure the bending stiffness and ultimate load to evaluate the biomechanical properties. Experimental data were expressed as mean ± SD.

### Micro-CT detection

Bone tissue was taken from the bone defect area on the eighth week after surgery for micro-CT detection, 3D reconstruction of the bone defect area, and quantitative analysis of bone mass and bone density. Specimens were collected and fixed with 4% paraformaldehyde for micro-CT analysis. Micro-CT scanning was performed by a Guantum GX microCT imaging system (Perkin Elmer, USA) with the following settings: acquisition, 36; voxel, 50 µm; reconnaissance, 25. The Guantum GX software was used for 3D reconstruction. Experimental data were expressed as mean ± SD.

### Statistical analysis

Statistical analyses were performed using Statistical Package for the Social Sciences (SPSS 19.0, IBM, New York, NY, USA). All data were expressed as the mean value ± standard deviation (SD). Statistical comparisons were conducted using analysis of variance (ANOVA) in which a *p*-value of less than 0.05 was considered statistically significant.

## Results

### Characterization of hydrogels

**FIGURE 1:**
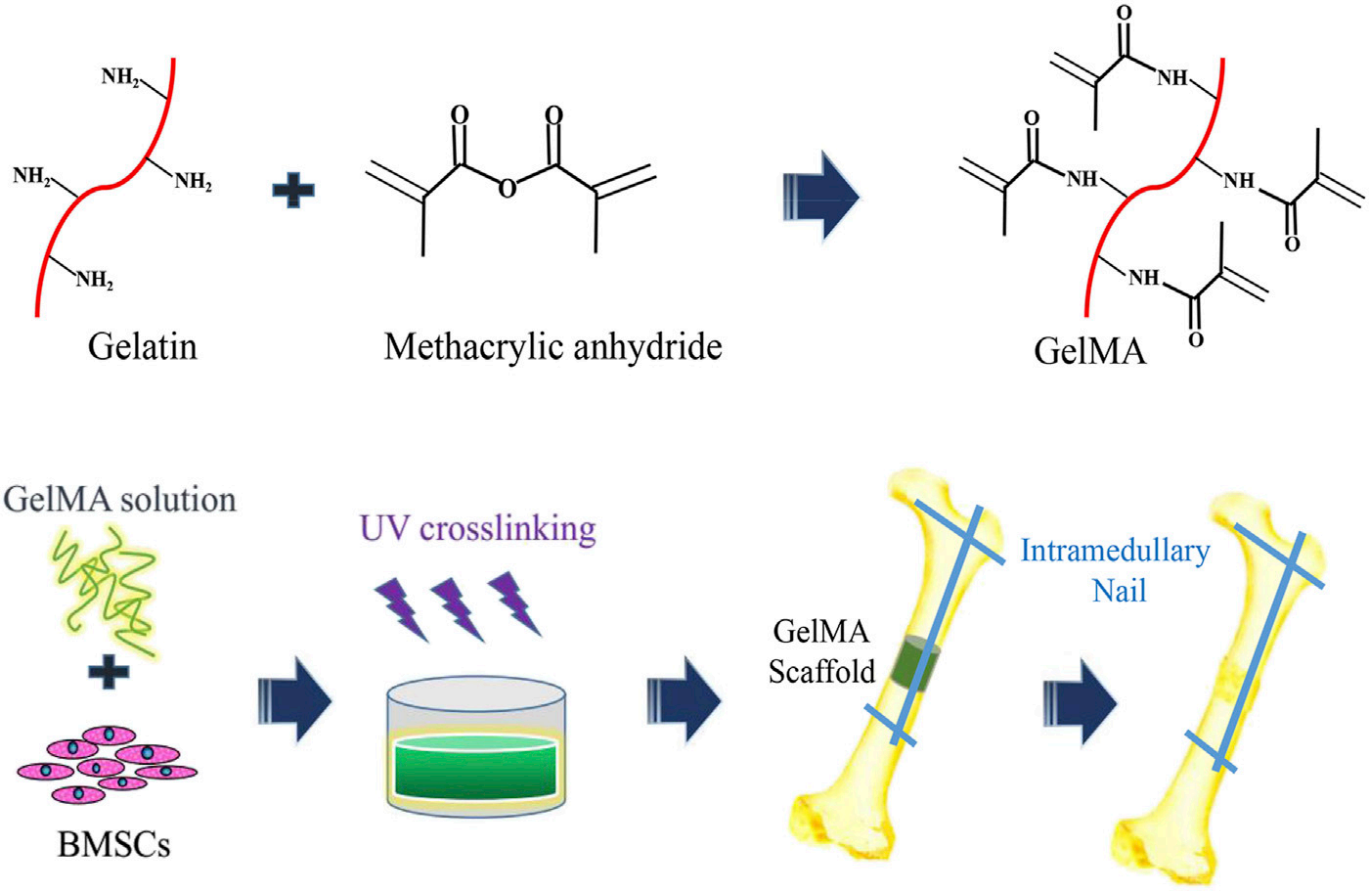
The schematic diagram of preparation of three-dimensional (3D) bone repair scaffolds supported by hydrogel cells and segments of bone graft defects.

**FIGURE 2:**
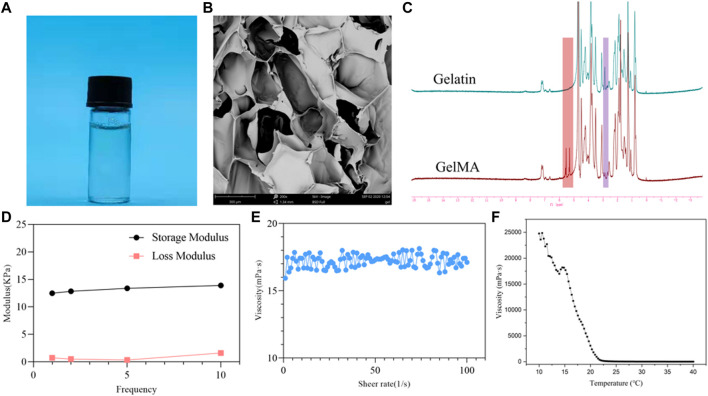
physicochemical properties of the methacrylated gelatin (GelMA) hydrogel. **(A)** GelMA solution; **(B)** scanning electron microscope (SEM) image of the GelMA hydrogel; **(C)** 1H-MR spectra of GelMA and gelatin; **(D)** DMA analysis of GelMA hydrogel with frequency dependency; **(E)** shear thinning of GelMA solution; **(F)** the viscosity of GelMA solution from 40°C to 10°C.

The overall process of this study is shown in Figure 1. As shown in Figure 2A, the GelMA can be rapidly dissolved in deionized water. After freeze drying of the GelMA photocured hydrogel, the porous structure of the hydrogel was observed by scanning electron microscope (SEM), which can be seen in Figure 2B. As can be seen from Figure 2C, the proton peak of methacrylic acid can be observed in the range of 5–6 ppm, which indicates that the GelMA has been successfully synthesized. In addition, in the range of 2.8–2.95 ppm, it was found that compared with the lysine proton peak of gelatin, the lysine proton peak of the GelMA was significantly weakened, indicating that the target reaction amino acid was consumed. The methacrylate substitution degree of the GelMA was calculated to be 87.6%. As shown in Figure 2D, the storage modulus of the GelMA hydrogel reaches 14.22 kPa at 10 Hz, and this mechanical strength can maintain the stability of the scaffold. In Figure 2E, as the shear rate increases from 0 to 100 s^−1^, the viscosity of the GelMA solution decreases gradually and remains stable, exhibiting shear thinning properties, which indicate that the GelMA is injectable. In Figure 2F, when the temperature decreases from 40°C to 10°C, the viscosity of the GelMA solution increases rapidly below 22°C to the point of physical gel formation.

### Biocompatibility of hydrogels

**FIGURE 3:**
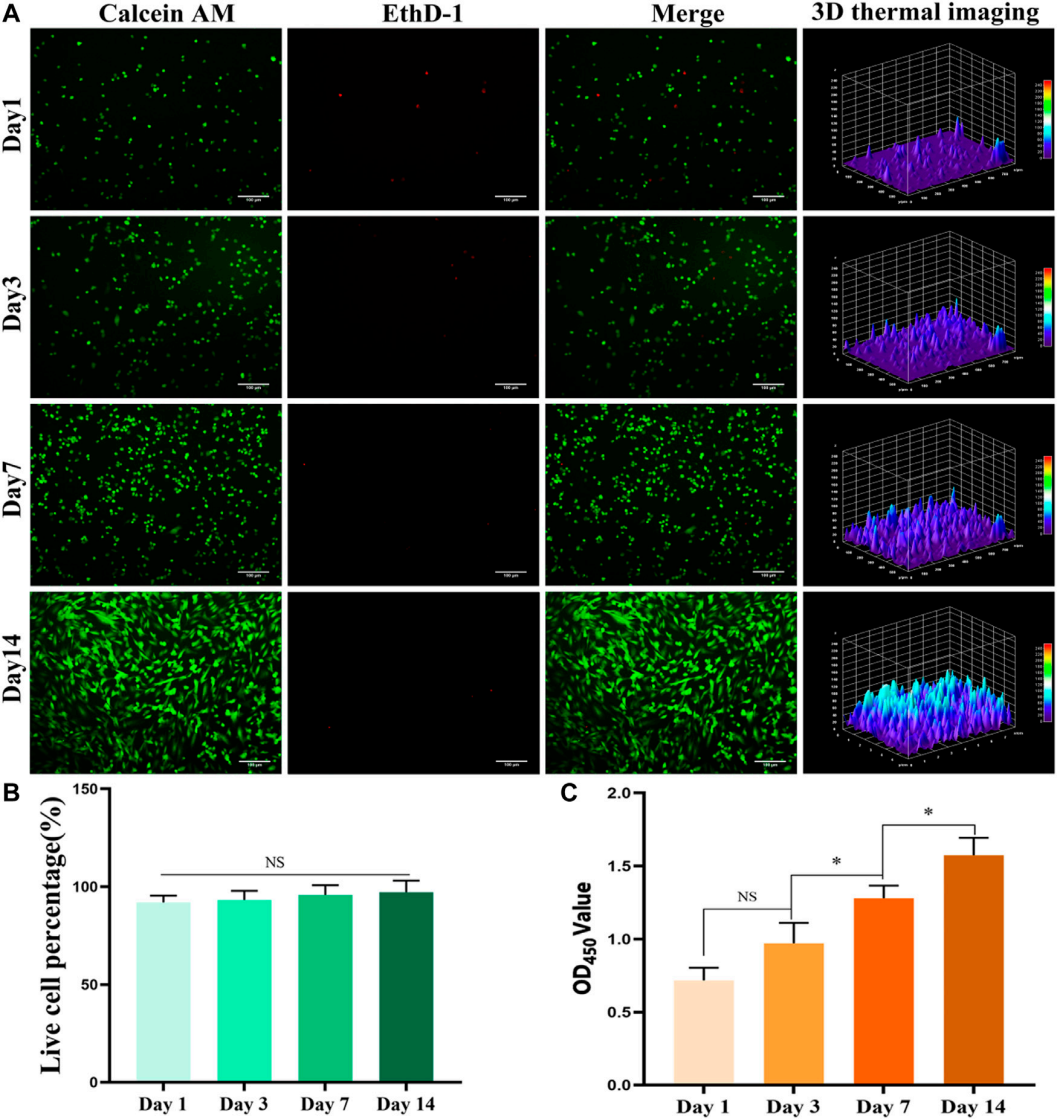
*In vitro* biocompatibility testing. **(A)** Live/dead staining result of bone marrow mesenchymal stem cells (BMSCs) in the hydrogel scaffold (bar = 100 μm). **(B)** The percentage of BMSCs living cells in the total number of cells in the hydrogel scaffold. **(C)** Results of cell activity and cytotoxicity tests of BMSCs in the hydrogel scaffold for 1, 4, 7, and 14 days. *p < 0.05.

The staining results of BMSCs with live/dead cells in the GelMA hydrogel bone repair scaffolds containing BMSCs are shown in Figure 3A (fluorescence staining of live/dead cells) and Figure 3B (percentage of the number of live cells in the total number of cells). Within 1–3 days of culture, the cells were spherical under the influence of a low adherent matrix. Over time, the cells increased and stretched, compared with the first day. Three-dimensional (3D) thermal imaging showed that fluorescence intensity increased over time. The staining results of live/dead cells showed that the survival rate of BMSCs in BMSC-loaded GelMA hydrogel bone repair scaffolds was high. After 1, 3, 7, and 14 days of culture, the number of living cells in the scaffold gradually increased, and the percentage of living cells exceeded 90%. In cell activity and toxicity tests performed on days 1, 4, 7, and 14, the results showed that the OD value increased with time (Figure 3C). The biocompatibility testing results showed that the GelMA hydrogel scaffold had good cell compatibility, and BMSCs could proliferate well in the scaffold.

### *In vivo* repair of large-segment bone defect using the bone marrow mesenchymal stem cell-laden methacrylated gelatin hydrogels

**FIGURE 4:**
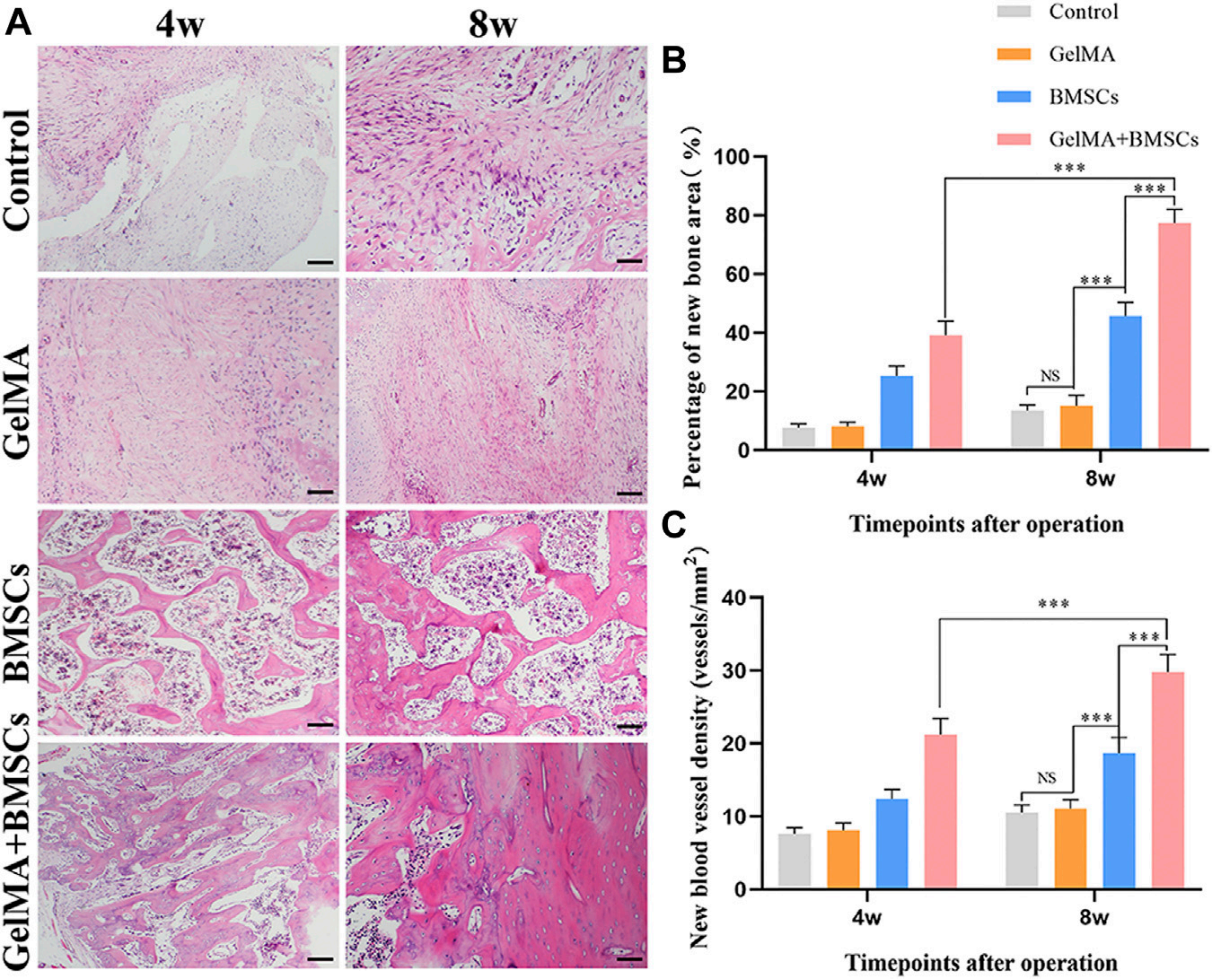
*In vivo* sample staining results. **(A)** Histological analysis of bone defects repaired by each group at weeks 4 and 8 after surgery (bar = 200 μm). **(B)** The percentage of the new bone area and **(C)** the density of neovascularization in the repaired bone defect area in each group at weeks 4 and 8 after surgery. ***p < 0.001.

To investigate whether the BMSC-loaded GelMA hydrogel bone repair scaffold can promote bone regeneration in the defect area, histological analysis was performed at weeks 4 and 8, respectively. Figure 4 shows the HE staining results of bone tissue in the bone defect area. As can be seen from Figure 4A, bone growth in the bone defect area in the BMSC group and the BMSC-carrying GelMA hydrogel scaffold group was vigorous at the fourth and eighth weeks after surgery, and new blood vessels were observed in the regenerated bone area. At the same time, compared with the BMSC group, the BMSCs-containing GelMA hydrogel scaffold group had more new bone formation and more mature tissue structure. In contrast, in the model control group and the GelMA hydrogel scaffold group, only a small amount of new bone formation occurred in the bone defect area, accompanied by more fibrous connective tissue formation.

Image Pro-Plus 6.0 software was used for quantitative analysis of the new bone and new blood vessels. The percentage of the new bone area was calculated according to the new bone area/total defect area × 100%, and the density of new blood vessels was measured according to the number of new blood vessels/bone defect area. Figures 4B **,** C show that the number of new bones and the density of new blood vessels increased in each group from weeks 4 to 8. The number of new bones and density of new blood vessels in the BMSC group was significantly higher than those in the model control group and GelMA hydrogel scaffold group at each time point (*p* < 0.01). However, the number of new bones and density of new blood vessels in the BMSC-carrying GelMA hydrogel scaffold group were significantly higher than those in the BMSC group (*p* < 0.05). The results show that the GelMA scaffold has the ability to promote the growth of new bone, and new blood vessels, that is, it has a good ability to promote bone regeneration.

**FIGURE 5:**
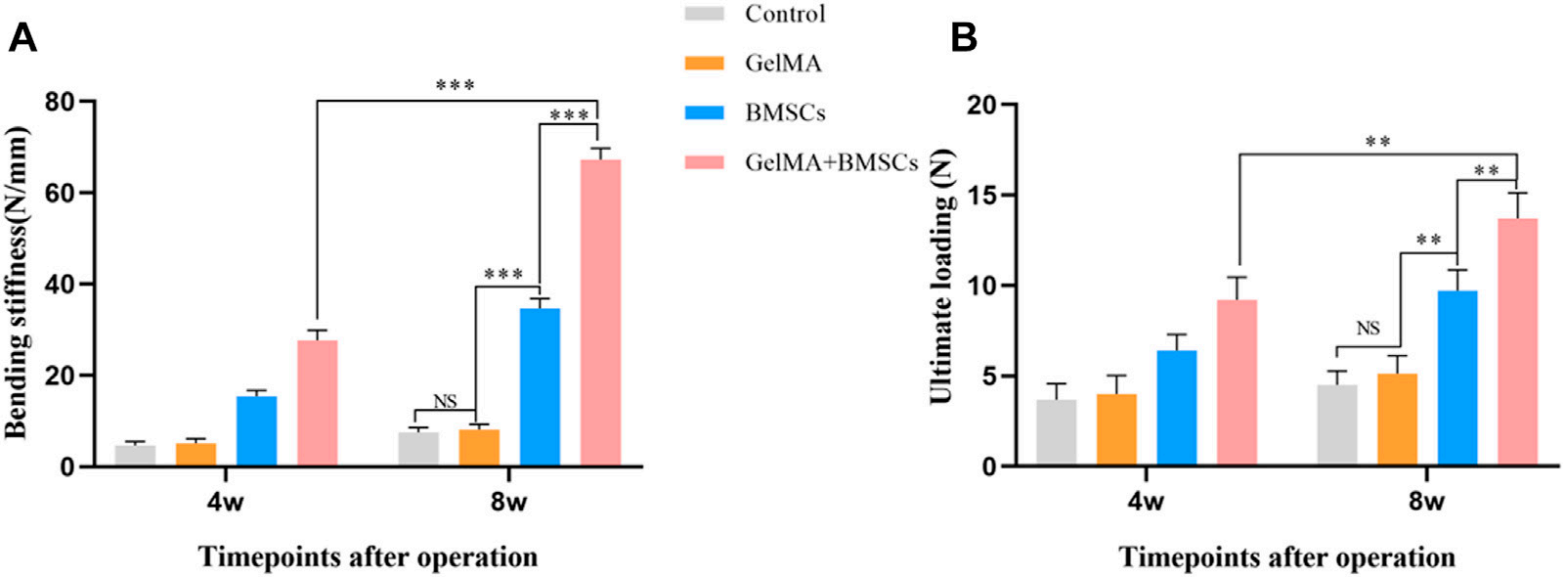
The biomechanical property test result. **(A)** The flexural stiffness and **(B)** the ultimate load of the bone defect repaired by each group at weeks 4 and 8 after surgery. **p < 0.01, ***p < 0.001.

The biomechanical property test results are shown in Figure 5. According to Figures 5A **,** B, at the fourth week after surgery, the bending stiffness and ultimate load of the BMSC-loaded GelMA hydrogel scaffold group were significantly higher than those of the BMSC group (*p* < 0.05), the model control group, and the GelMA hydrogel scaffold group (*p* < 0.01). There was no statistically significant difference between the model control group and the GelMA hydrogel scaffold group (*p* > 0.05). The biomechanical performance at week 8 was similar to that at week 4, and the difference in bending stiffness and ultimate load between the BMSC-containing GelMA hydrogel scaffold group and the BMSCs group was more significant (*p* < 0.01). The experimental results show that the BMSC-loaded GelMA hydrogel bone repair scaffold can improve the mechanical strength of the defect tibia.

**FIGURE 6:**
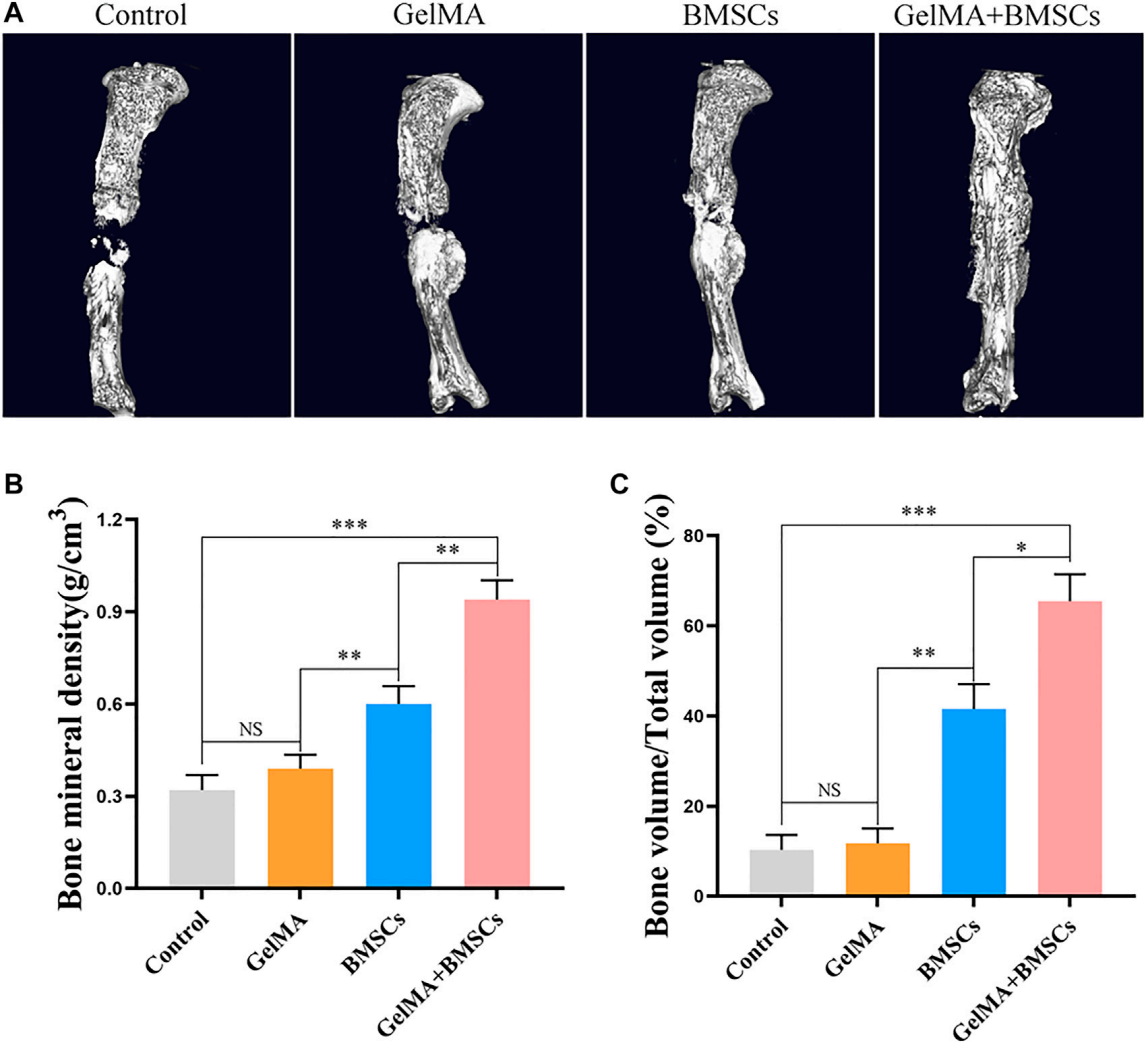
CT detection results. **(A)** The micro-CT 3D reconstruction models of bone defects repaired by each group at the eighth week after surgery. **(B–C)** The bone density and bone volume of each group at 8 weeks after surgery. **p < 0.01, ***p < 0.001.

Micro-CT detection was performed on the bone defect area at the eighth week after surgery, and three-dimensional reconstruction was conducted. Figure 6A (micro-CT 3D reconstruction model) shows that the bone bridge and callus formation in BMSC-carrying GelMA hydrogel scaffold was significantly better than that in the BMSCs group, GelMA hydrogel scaffold group, and model control group. The quantitative results of bone mass (Figure 6B) and bone mineral density (Figure 6C) were consistent with the above: the mean bone mass and bone mineral density of the BMSC-loaded GelMA hydrogel scaffold group were significantly higher than that of the BMSC group alone, the GelMA hydrogel scaffold group, and the control group (*p* < 0.01). There was no statistically significant difference between the GelMA hydrogel scaffold group and the control group (*p* > 0.05).

## Discussion

The repair of the large segmental bone defect is still a difficult problem in orthopedic clinical treatment (Wojda and Donahue, 2018). When the defect exceeds the critical size, self-healing cannot be achieved (Schemitsch, 2017). Autografts or allografts are often used to fill defects, but are limited by factors, including infection, immune response, and infectious diseases (Roseti et al., 2017). The development of bone tissue engineering shows promise in repairing large segmental bone defects (Kim et al., 2017). Hydrogels are hydrophilic polymers that are insoluble in water. After soaking in water, the weight of the hydrogel increases to several times its original dry weight (Lu et al., 2018). Hydrogels have been widely used in tissue engineering in recent years because the internal structure and composition of hydrogels are similar to the ECM (Xu et al., 2018). Hydrogels have the potential to mimic ECM in transporting nutrients and metabolites and providing an appropriate microenvironment (Ngo et al., 2018). A variety of natural and synthetic hydrogel polymers have been developed, including gelatin, alginate, fibrin, chitosan, hyaluronic acid (HA), polyethylene oxide (PEO), and polyethylene glycol (PEG) (Yang et al., 2017). Among them, GelMA-based hydrogels show great potential due to their biocompatibility and mechanical stability. Proper internal porosity is necessary for tissue-engineered materials to maintain cell growth and nutrient transfer. The water-rich GelMA hydrogel mimics the cell matrix microenvironment, and the pore structure facilitates cell adhesion, proliferation, and growth (Shaunak et al., 2017).

In general, scaffolds should carry a mechanical load while promoting tissue regeneration (Biggemann et al., 2018; Zhang et al., 2019c). However, the mechanical properties of hydrogels are lower than that of bone tissue, so the use of hydrogels is usually limited to the areas with no or low load (Ho-Shui-Ling et al., 2018). This study confirmed that the mechanical properties of hydrogels were sufficient to support bone repair with intramedullary nails or plates. Furthermore, in this study, we detected the influence of temperature on GelMA hydrogel viscosity. The GelMA solution maintains low viscosity at room temperature and is favorable for injection. Compared with traditional prefabricated scaffolds, injectable hydrogels can fill defects of any size or shape without requiring additional surgical procedures and are easily formed by mixing with cells (Gupta et al., 2006; Ma et al., 2017).

Compared with traditional two-dimensional (2D) cell culture, three-dimensional (3D) cell culture systems better fit the physiological environment in terms of cell–cell and cell–matrix interaction and diffusion behavior (Booij et al., 2019). In 2D cell culture, the signaling molecules released by the cells are immediately diluted in a relatively larger volume of cell culture medium (Di Modugno et al., 2019). In addition, physiological gradients of signaling molecules, metabolites, and oxygen cannot be generated in 2D culture systems, whereas 3D culture can better study signaling, nutrition, and metabolism in a concentration- and site-dependent manner (Ravi et al., 2015; Xing et al., 2020). MSCs are easily extracted from bone marrow, fat, and synovium (Le et al., 2020). MSCs can be differentiated into a variety of cell lines for specific biomedical applications (Han et al., 2019). As an important MSCs-specific characteristic, differentiation potential affects the fate of MSC. MSCs from different tissue sources show different differentiation trends. Compared with adipose-derived MSCs, BMSCs show stronger osteogenic ability (Han et al., 2019). In this study, BMSCs were coated with hydrogel in a 3D environment to detect the biocompatibility of GelMA *in vitro*. BMSCs have a strong ability of regeneration and differentiation. Cell therapies using BMSCs are currently involved in more than 900 clinical trials (Kirsch et al., 2019; Wang et al., 2021). The fate of BMSCs is influenced by the microenvironment provided by the injection of hydrogels after transplantation. As an ECM analog, the injected hydrogel can affect the migration, proliferation, differentiation, and intercellular communication of stem cells (Chen et al., 2012; Ma et al., 2017). In this study, BMSCs could grow and proliferate in the GelMA hydrogel by means of *in vitro* culture, which proved that the GelMA hydrogel had good biocompatibility. In the *in vivo* experiment, with the fixation of intramedullary nails, the GelMA hydrogel-wrapped BMSCs were implanted into the bone defect site to verify its osteorepair ability. The BMSC-loaded GelMA hydrogel group was significantly superior to the other groups in both morphological and mechanical results. HE results showed that the cell-loaded GelMA hydrogels not only promoted bone regeneration but also correspondingly promoted blood vessel regeneration, and abundant blood supply is the basis of tissue regeneration.

Due to the limitation of the mechanical properties of GelMA, GelMA is mostly used to study non-load-bearing bone (such as skull) defects or simulated periosteum (Gonçalves et al., 2021; Xiang and Cui, 1186). In these studies, the GelMA hydrogel quickly restored the integrity of the damaged bone surface. Interestingly, compared with other commonly used filling materials, such as metal and ceramic, GelMA can deposit extracellular matrix and type II collagen, which is more conducive to blood vessel and nerve regeneration (Benmassaoud et al., 2020; Xiang and Cui, 1186). Consistent with literature reports, in this study, the number of new vessels in the hydrogel stent group loaded with BMSCs was significantly higher than that in the other groups. In the repair of segmental bone defect, the two ends of the defect are completely disconnected, and the bone marrow cavity is completely exposed, so the biocompatibility of the repair material is highly required. Therefore, in the previous study, the lacunar bone defect model was used in the *in vivo* experiment of the composite scaffolds, and the repair of segmental bone defects by composite scaffolds is prone to infection and osteonecrosis (Wang et al., 2021; Zhang et al., 2021). In the segmental bone repair model in this study, the biocompatibility advantage of the GelMA was demonstrated without bone infection. In future research, the hydrogel scaffold will be optimized from the aspects of material and technology. First, to add osteogenic components, such as magnesium ion, lithium ion, and nano-hydroxyapatite. Some reports have demonstrated that hydrogels loaded with magnesium, lithium, or hydroxyapatite have enhanced mechanical strength and osteogenic induction (Wang et al., 2020a; Wang et al., 2020b; Zhu et al., 2020). Second, microneedle injection technology can be introduced to increase accuracy and efficiency (Lee et al., 2020).

In conclusion, the BMSC-carrying GelMA hydrogel scaffold has good mechanical properties and biological compatibility. Implantation of bone defects can effectively promote the regeneration of bone and blood vessels, improve the mechanical strength of bone defects, and effectively promote the repair of bone defects. It has a good ability to promote bone regeneration and has great potential for application.

## Data Availability

The raw data supporting the conclusions of this article will be made available by the authors without undue reservation.
